# Effect of Metal Cocatalysts and Operating Conditions on the Product Distribution and the Productivity of the CO_2_ Photoreduction

**DOI:** 10.1021/acs.iecr.1c02514

**Published:** 2022-02-18

**Authors:** Francesco Conte, Alberto Villa, Laura Prati, Carlo Pirola, Simona Bennici, Gianguido Ramis, Ilenia Rossetti

**Affiliations:** † Dip. Chimica, Università degli Studi di Milano, via C. Golgi 19, 20133 Milan, Italy; ‡ Institut de Science des Matériaux de Mulhouse (IS2M), Université de Haute-Alsace, CNRS, IS2M UMR 7361, F-68100, Mulhouse, France; § Dip. Ing. Chimica, Civile ed Ambientale, Università degli Studi di Genova and INSTM Unit Genova, via all’Opera Pia 15A, 16145 Genoa, Italy

## Abstract

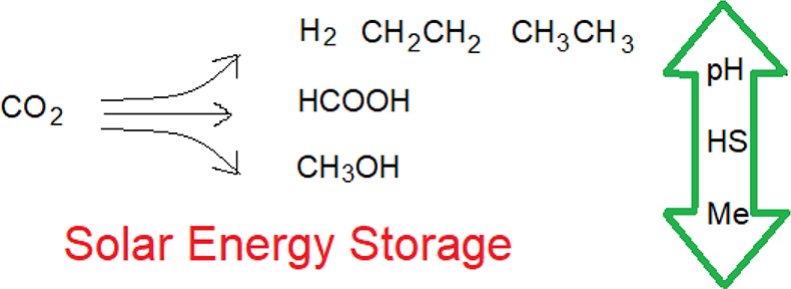

The CO_2_ photoreduction is a promising way to convert one of the most abundant greenhouse gases to valuable chemicals. The photoreduction in the liquid phase is limited by the low solubility of CO_2_ in water, but this point is overcome here by using an innovative photoreactor, which allows one to work up to pressures of 20 bar, improving the overall productivity. The photoreduction was performed in the presence of Na_2_SO_3_ and using in primis commercial titanium dioxide (P25) and a set of titania catalysts functionalized by surface deposition of either monometallic or bimetallic cocatalysts. The gaseous products were hydrogen and traces of CO, while, in the liquid phase, formic acid/formate, formaldehyde and methanol were quantitatively detected. The pH was observed to shift the products distribution. A neutral environment led mainly to hydrogen and methanol, while, at pH 14, formate was the most abundant compound. The trend for monometallic cocatalysts showed enhanced productivity when using noble metals (i.e., gold and platinum). In order to limit the cost of the catalytic material, bimetallic cocatalysts were explored, adding titania with Au+Ag or Au+Pt. This may open to the possibility of performing the reaction with a smaller amount of the most expensive metals. In the end, we have expressed some conclusions on the cost of the photocatalysts here employed, to support the overall feasibility assessment of the process.

## 1 Introduction

Warnings on climate change are unequivocal, and since the 1950s, many of the observed phenomena are unprecedented over decades to millennia leading to a globally averaged warming of 0.85 °C over the period 1880–2012, as explained in the fifth assessment report of IPCC.^1^ Among the effects described there, one can mention the following: higher frequency of extreme climate events, glacier shrinkage, sea-level increase, drought with related fires, and oceans acidification. Equally unequivocal is the human contribution to the phenomenon, as a consequence of greenhouse gases emissions.^1^ In particular, the atmospheric concentration of carbon dioxide, methane, and nitrous oxide has increased to levels unprecedented in at least the last 800 000 years. CO_2_ concentration has increased by 40% since preindustrial times, primarily from fossil fuel emissions and secondarily from net land use change emissions.^1^ Limiting CO_2_ levels is important not only to reduce emissions by decreasing the use of fossil fuels, but also to adopt strategies to remove CO_2_ using the approach of carbon capture and sequestration (CCS) from point sources.^2^ CCS has some drawbacks,^3^ such as the risk of leakage, energy consumption during compression and transportation, and null direct economical return.

A smarter alternative to the storage of CO_2_ is to use CO_2_, either directly (e.g., as a solvent, working fluid, or heat transfer fluid) or as a reagent (to convert it to new/regenerated products). The latter option is harder than the use of CO_2_ itself, since carbon dioxide is a very stable compound and it requires harsh conditions (both high temperature and pressure) to be converted to useful chemicals, but undoubtedly better, since it converts a waste to a resource in a fully circular way.

Unconventional strategies must be then adopted to make CO_2_ conversion feasible. Several studies on CO_2_ photoreduction have already been performed.^4−6^ They usually consist of the use of a photocatalyst that allows CO_2_ to react under milder conditions than those needed through thermocatalytic activation. These methods represent a valid green and sustainable alternative, especially when using sunlight as a photon source and water as a solvent, electron donor, and proton supplier. One of the most studied photocatalysts is TiO_2_, both in the rutile and anatase form. Titanium dioxide is a semiconductor, which has the great advantage of being resistant to photocorrosion, nontoxic, and inexpensive.^7^ Moreover, its production and recyclability are well-established.^8^

Unfortunately, the reaction has some criticisms, which limit its applicability at the point that currently no fully feasible solution exists.^9,10^ Some key points are listed below:

(1) Solar light harvesting must be improved. TiO_2_ has a bad gap between 3.2 eV (anatase) and 3.05 eV (rutile), which allows photons to be absorbed from the ultraviolet region, only a minor fraction of the solar spectrum.
(2) The charge recombination ratio must be reduced. The semiconductor can effectively promote a reaction if generated electrons and holes survive enough times to reach the surface of the catalyst and react with the adsorbed reactants.
(3) The CO_2_ solubility in the working solvent must be enhanced.^11^ Generally, water is used (inexpensive and green solvent) but limited CO_2_ solubility limits the reaction rate; increasing the pressure could allow enhanced solubility and surface adsorption with consequent higher productivity.

In this work, the first two issues were addressed by deposition of metallic (Cu, Ag, Au, Pt)^12−14^ cocatalysts over the TiO_2_ surface, while the third one is strictly related to the reaction conditions, such as pressure and pH. Generally, metal particles form an electrical connection with TiO_2_ and may trap photogenerated electrons in case of a Fermi level placed above the one of the semiconductors (Schottky barrier).^15^ The holes accumulate on the catalyst. Moreover, working in the presence of a hole scavenger (HS), either organic or inorganic, prevents the accumulation of holes, reducing the ratio of recombination and increasing the productivity.^10,16,17^

The answer to issue 3 is increasing the operating pressure to improve the solubility of CO_2_ in water. All the reactions were thus performed with an innovative photoreactor,^9,18−20^ which allows one to work at pressures up to 20 bar. In this work, the results of CO_2_ photoreduction tests with a wide range of titania-based catalysts, including monometallic and bimetallic promoted samples, are reported. The effect of the reaction parameters (i.e., pressure, HS conversion, and pH) was explored as well.

## 2 Experimental Section

### 2.1 Materials Preparation

P25 is a commercial nanometric titanium oxide supplied by Evonik.

Au 0.1, 0.2, and 0.5 wt %/P25, prepared via a deposition–precipitation (dp) technique in very dispersed form, according to a recipe reported elsewhere,^19^ were tested in our preliminary investigations, demonstrating the highest activity for this reaction for 0.2–0.5 wt % (corresponding to 0.055–0.15 mol %).^19^

Au 0.2 mol %/P25wi (where wi denotes= wet impregnation, to distinguish this Au-loaded samples from those prepared by dp), Cu 0.2 mol %/P25 and Pt 0.1 mol %/P25 were prepared by wet impregnation. Shortly, the desired amount of titania and metal precursor (see Table 1) is added to a round flask and covered with distilled water. A suspension is formed through stirring (2 h) and then the solvent is removed via evaporation under reduced pressure. The resulting powder is collected and dried overnight into an oven (105 °C) and then reduced to metallic form in a tubular oven under hydrogen flux at temperatures specified in Table 1 and selected after a preliminary temperature-programmed reduction (TPR) experiment. The temperature was set according to the temperature of reduction and accounted also for a partial titania reduction through formation of oxygen vacancies.

**Table 1:** Catalyst Recipes for Wet Impregnation Technique^a^

precursor	gold(III) chloride (purity, >99.99%)	platinum acetylacetonate (purity, >99.0%)	copper(II) acetate monohydrate (purity, >97%)	silver nitrate (purity, >97%)
metal loading (mol %)	0.2	0.1	0.2	0.1
mass ratio (mg/g_TiO_2__)	3.44	10.15	4.69	13.15
heating ramp (°C/min)	5	5	5	5
reduction temperature (°C)	700	700	500	150
reduction time (min)	180	180	180	180

a Precursors provided by Sigma–Aldrich.

Bimetallic alloys were also used as cocatalysts. Au_*x*_Ag_*y*_ 1 wt %/P25 and Au_*x*_Pt_*y*_ 1 wt %/P25 were prepared through colloidal-immobilization synthesis. In the first case, 400 mL of distilled water AgCl (10 mg Ag/mL) (Fisher Scientific), HAuCl_4_ (10 mg/mL) water solution, and 0.10 mL of a water solution of poly(vinyl alcohol) 87%–90% hydrolyzed (PVA) (1 wt %) were added to a flask under vigorous stirring. Then, 10 mL of NaBH_4_ 0.1 M solution was added to the flask (NaBH_4_/metal = 4 mol/mol metal). The resulting colloidal solution was maintained under vigorous stirring for 10 min to stabilize the nanoparticles formed during the reduction process. After that, P25 (0.99 g) and 0.5 mL of sulfuric acid (98%, Sigma–Aldrich) were added dropwise and stirred for 1 h to allow complete immobilization of metal nanoparticles on the solid support. The catalyst was then filtered under vacuum and washed with distilled water. The resulting powder was dried in an oven at 105 °C for 4 h. The procedure followed for Au_*x*_Pt_*y*_ 1 wt %/P25 was analogous, but a NaBH_4_/metal ratio of 8 is used and no sulfuric acid was added.

### 2.2 Materials Characterization

X-ray diffraction (XRD) analyses were performed by the Rigaku D III-MAX horizontal-scan powder diffractometer (Tokyo, Japan) using Cu Kα radiation with a graphite monochromator on the diffracted beam.

N_2_ adsorption and desorption isotherms of samples were collected with a Model ASAP2020 apparatus (Micromeritics, Norcross, GA, USA). The Brunauer–Emmett–Teller specific surface area (BET SSA) and pore volume have been calculated from N_2_ adsorption/desorption isotherms, collected at −196 °C for the samples previously outgassed at 150 °C for 4 h. Micropore volume was calculated according to the *t*-plot method.

Diffuse reflectance (DR) UV–vis spectra of samples were measured on a Cary 500 UV–vis NIR spectrophotometer (Varian Instruments, Santa Clara, CA, USA) in the range of 200–800 nm.

Scanning electron microscopy (SEM) and energy-dispersive X-ray spectroscopy (EDS) have been performed on a Model JSM-7900F Schottky field emission scanning electron microscope (JEOL, Tokyo, Japan), by applying an accelerating potential of 20 kV.

X-ray photoelectron spectroscopy (XPS) was performed on a Model SES-2002 (VG SCIENTA) spectrometer (energy resolution of 0.4 eV).

### 2.3 Photoreactor and Testing Conditions

Photocatalytic activity tests were conducted using an innovative pressurized batch photoreactor, which has been described in detail elsewhere.^21,22^ Briefly, it is a cylinder-shaped reactor surrounded by a jacket where water can circulate and equipped with a quartz window, which allows the introduction of a coaxial lamp. The reactor is designed to work under pressures up to 20 bar and temperatures up to 90 °C, thanks to a thermostatic system. Internal volumes of ca. 1.3 and 1.2 L of solution were used for each experiment, with ca. 0.1 L head space for gas accumulation and sampling. Catalyst dispersion is ensured by a magnetic stirrer set at 400 rpm and placed under the reactor. The photon source is a medium-pressure 125 W Hg vapor lamp made of two bulbs, which emits in the range of 254–364 nm. The measured average irradiance was 120 W/m^2^ in the UVA range.

The optimal catalyst concentration and catalyst/HS ratio was found in a previous work.^16^ Na_2_SO_3_ was used as hole scavenger and negligible CO_2_ photoreduction has been observed without its addition. The catalyst and the HS have been loaded with distilled water. The solution was allowed to saturate with CO_2_ at the desired pressure and room temperature for one night and the resulting pressure was taken as a reference. The reaction started when the reactor reached 80 °C (measured with a thermocouple) and the lamp was switched on. All of the reported results were collected after 24 h (if not specified otherwise) or 3–6 h irradiation, as specified, to limit gas-phase products and organics consumption in the liquid phase.

Starting composition and liquid products have been analyzed via the withdrawal of three samples of 10 mL of solution at the beginning and the end of the reaction. To analyze the liquid products, a high-performance liquid chromatography (HPLC) system (LC-4000 series, Jasco) equipped with the proper column (2000–0 BP-OA, Benson Polymeric), equipped with both UV (UV-4074, Jasco) and refractive index (RI-4030, Jasco) detectors, have been used. Aqueous H_2_SO_4_ solution (1 mmol/L) was used as eluent. The gas products were collected in the headspace of the photoreactor and analyzed by a gas chromatograph (7820, Agilent, Palo Alto, CA, USA) equipped with a TCD detector with the proper set of configurations for the quantification of H_2_, CH_4_, and both polar and nonpolar light gases. The analyses of the liquid- and gas-phase products were revealed to be reproducible within a maximum error of 4%. Broader excursion was observed for the overall testing results, with average error of ca. 6.5%, maximum 10%. The issues for reproducibility are attributed to mixing of the catalyst, especially when very low amounts are used, and to the sampling, which induces a pressure variation in the reactor with possible loss of products and transient operation.

The hole scavenger conversion was determined by means of iodometric titration: a selected amount of sample was mixed with a precise amount of potassium iodate water solution, then potassium iodide and diluted hydrochloric acid were added in excess. This mixture led to the production of free iodine, which was then titrated with sodium thiosulfate.

## 3 Results and Discussion

### 3.1 Materials Characterization

A summary of the main characterization results is reported in Table 2.

**Table 2:** Catalyst Properties, Obtained from N_2_ Sorption Isotherms at −196 °C; XRD Diffractograms and Band Gap Calculation from DR UV-Vis Data Elaborated According to Tauc Plots

sample	P25	Au 0.2 wt % /P25^26^	Au 0.2 mol %/P25wi	Pt 0.1 mol %/P25	Ag 0.1 mol %/P25	Cu 0.2 mol %/P25	Au_8_Ag_2_ 1 wt %/P25	Au_2_Ag_8_ 1 wt %/P25	Au_6_Pt_4_ 1 wt %/P25	Au_2_Pt_8_ 1 wt %/P25
phases (%)	A(78)+R(22)	A(78)+R(22)	A+R	A(87)+R(13)	A(70)+R(30)	A+R	A+R	A+R	A+R	A+R
BET surface area (m^2^ g^–1^)	45	55	18	55	57	6	–	–	16	–
crystallite size (nm)	15	15	–	18 (A), 28 (R)	18 (A), 28 (R)	–	16 (A), 23 (R)	16 (A), 24 (R	19 (A), 29 (R)	17 (A), 25 (R)
total pore volume (cm^3^ g^–1^)	0.11	0.27	0.11	0.024	0.36	0.05	–	–	0.20	–
t-plot micropore volume (cm^3^ g^–1^)	0.012	0.005	0.0047	0.0038	0.0045	0.0031	–	–	0.0038	–
BJH adsorption pore width (nm)	22	31	89	10	29	9	–	–	58	–
band gap (eV)	3.41	3.17	2.87	3.11	3.07	2.71	2.88	2.96	2.85	–

Commercial titanium dioxide (P25) and Au 0.2 wt %/P25 were widely characterized in a previous work.^16^

**Figure 1:**
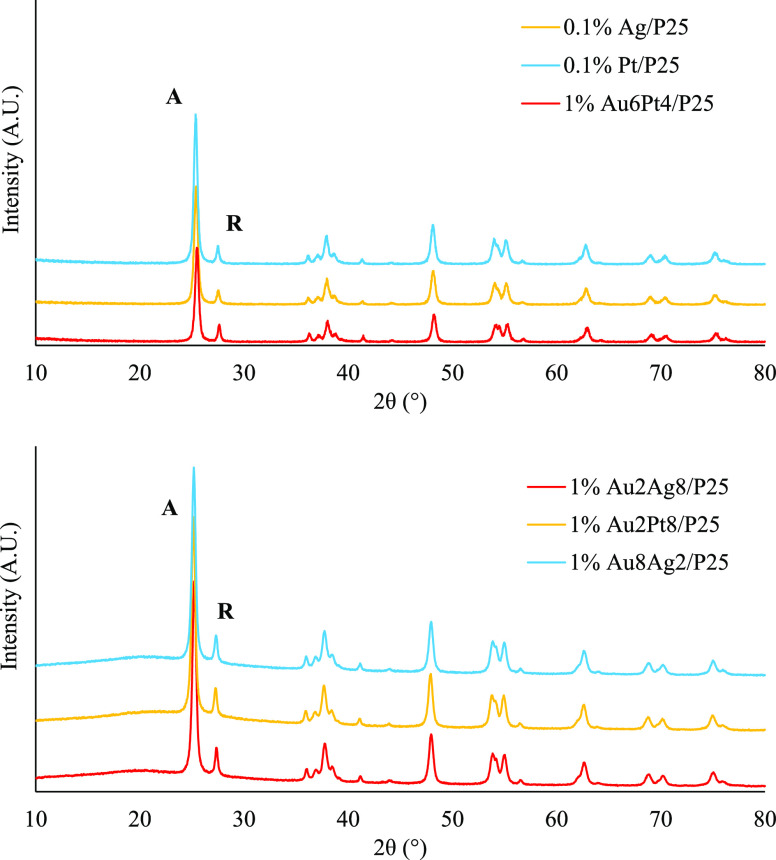
XRD spectra of some photocatalysts employed. “A” and “R” represent the anatase and rutile main reflections, respectively.

The XRD patterns reported in Figure 1 illustrate no major changes of the phase composition due to the metal deposition and post treatment. In addition, no peaks associated with the metallic phase appeared, which is expected since the metal represents only a small fraction of the entire particle weight and is very well dispersed over the titania surface. The phase composition was calculated according to the intensity ratio between the most intense peak of both anatase and rutile. The former was the major phase and it was usually found with percentage between 70% and 87%. The crystallite size of the deposited photocatalyst was found to be slightly higher than the benchmark P25, up to 20% in the case of the anatase phase and with rutile crystals, on average, bigger than those of anatase. Similar results have been achieved in the case of bimetallic photocatalysts.

**Figure 2:**
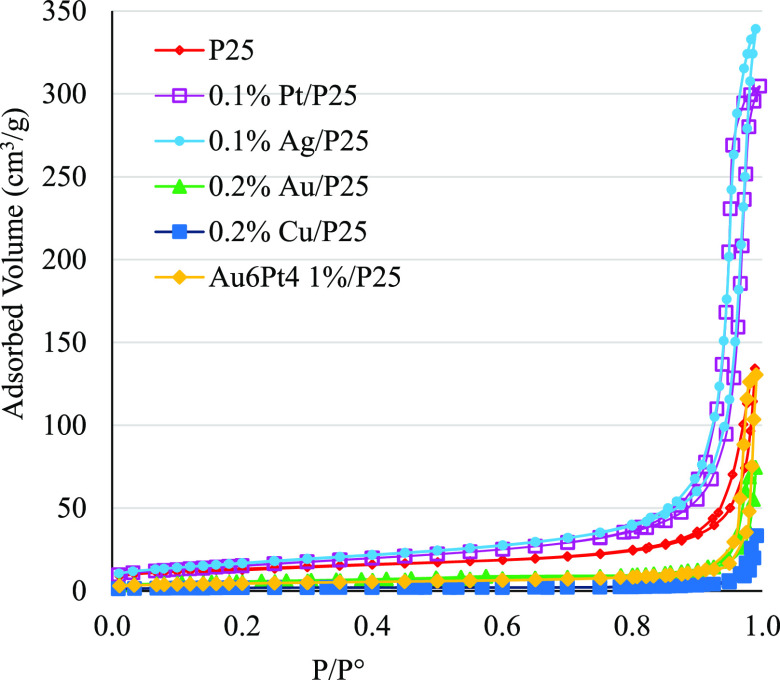
Nitrogen adsorption–desorption isotherms of selected metal-loaded photocatalyst, compared to the bare P25.

The N_2_ adsorption/desorption isotherms were Type 2 with a very small hysteresis loop. Limited porosity was evident, mainly due to the agglomeration of nanoparticles. The BET SSA and pore volumes were derived from the N_2_ adsorption/desorption isotherms, exemplified in Figure 2. The results are summarized in Table 2. Generally, catalysts obtained by wet impregnation did not show a significant reduction of the surface area, if compared to bare P25.^9,23^ By contrast, it is likely that the relatively high temperature used for calcination have caused some sintering for Cu 0.2 mol %/P25 and Au 0.2 mol %/P25wi samples, which were characterized by a smaller surface area.

In addition, Table 2 illustrates that the porosity of the functionalized catalyst was lower than that of P25 and, once more, this may be caused by the reduction at high temperature: indeed, the Pt and Cu samples exhibited the greatest loss, in terms of pore volume.

The band-gap energy (*E*_g_) of the catalysts was calculated according to the Tauc plot elaboration of DR-UV–vis spectra.^24^ As we expected, band-gap calculations showed a significant reduction of the *E*_g_ for all of the metal-deposited catalyst, with respect to bare P25 (*E*_g_ = 3.41 eV).^25^ The band gap of the semiconductor can be tuned for a given semiconductor by doping with cocatalysts. However, this is possible by incorporation of the heteroatom inside the crystal lattice, which is not expected under the current preparation conditions, which lead to surface deposition of the cocatalyst. The band gap in this case was mainly modified by the partial reduction of titania, occurring during activation in H_2_ at relatively high temperature and favored by the presence of the metal itself, which activates hydrogen more effectively, to reduce the oxide. We must stress that, in this work, *E*_g_ was not a strictly critical parameter, since the light source employed was an UV-type lamp able to excite the electrons of all the catalysts, but the future aim is to exploit directly sunlight so it may be interesting to test the materials with the lowest band gap, such as Cu 0.2 mol % (*E*_g_ = 2.71 eV). Therefore, the role of the metal was predominantly the sequestration of the photogenerated charges, rather than the modification of the optical properties.

**Figure 3:**
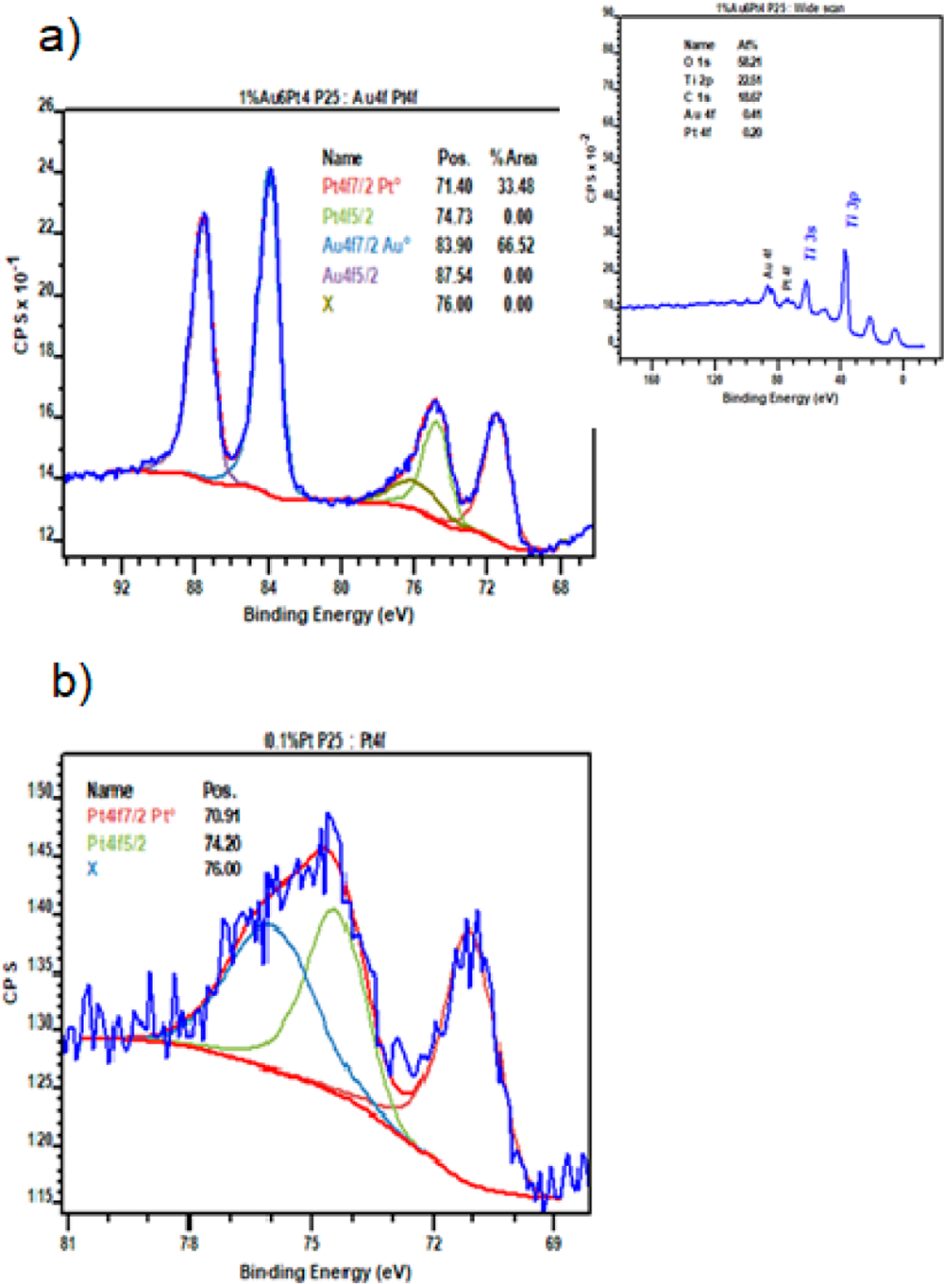
XPS spectra of samples (a) 0.1 mol % Pt and (b) 1 wt % (Au6Pt4).

XPS spectra were collected on some representative samples and reported in Figure 3. The spectra confirm the surface composition of the samples and the metallic oxidation state of both Pt and Au. This was true, irrespective of the deposition method.

**Figure 4:**
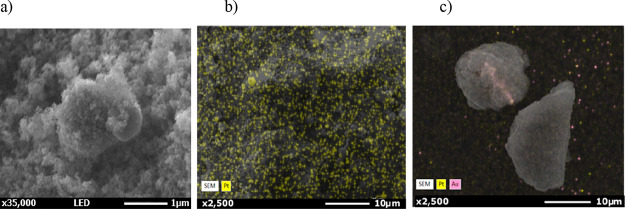
(a) SEM micrograph and (b) EDS map of the 0.1 mol % Pt sample; (c) EDS map of sample 1 wt % (Au6Pt4).

The morphology of the samples was observed through SEM and the particle size of P25 (ca. 20 nm) was retained even after calcination at 700 °C (see, e.g., Figure 4a). EDS maps are reported for two representative monometallic and bimetallic samples, with similar results for the other catalysts. High surface dispersion of Pt was achieved notwithstanding the calcination at high temperature and results were similar to that obtained under milder conditions with the bimetallic Au/Pt samples (Figures 4b and 4c).

### 3.2 Photocatalytic Activity for the Reduction of CO_2_

#### 3.2.1 Effect of pH

At first, a blank test was conducted in the absence of lamp irradiance. Being that the reaction is photocatalytic, we expected no products of reduction and the results confirmed the hypothesis.

The photoreduction involves multistep electron additions and various intermediates, depending on the type of photocatalysts used and reaction conditions. This complexity leads to a broad spectrum of products, e.g., CO, H_2_, HCOOH, HCHO, CH_3_OH, through a mechanism discussed previously.^20,27,28^

**Figure 5:**
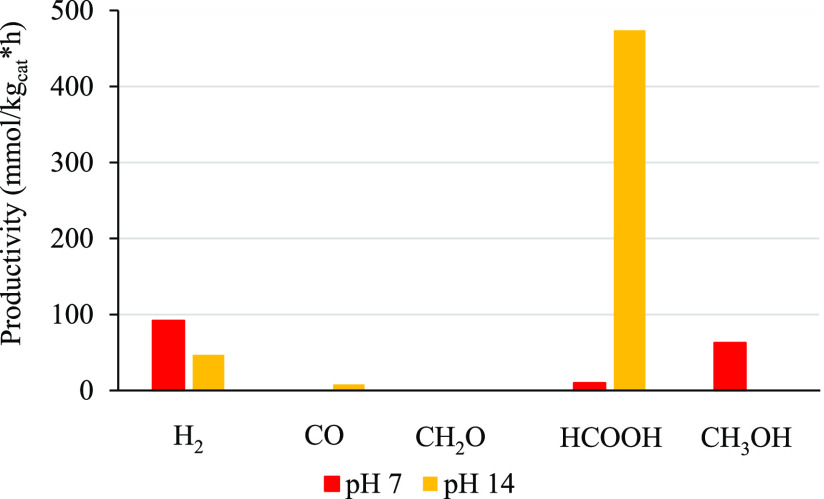
Effect of pH on product distribution and productivity, using Au 0.2 wt %/P25 at 18 bar, 0.6 g/L of catalyst, and 0.85 g/L of HS.

The products distribution and the productivity has been determined at pH 7 and 14, with a catalyst concentration of 0.6 g/L and 0.85 g/L of HS over Au 0.2 wt %/P25. The results are reported in Figure 5. The overall CO_2_ conversion increased at basic pH, with a shift from gaseous toward liquid products (mainly formate). A possible explanation of the enhanced productivity, independently from the type of products obtained, is that a basic solvent improves CO_2_ solubility and leads to formation of CO_3_^2–^ and HCO_3_^–^, which may be subsequently reduced to formaldehyde or formate. Indeed, at pH 14, the main species present is carbonate, which is consecutively transformed to adsorbed CO as explained in ref (21). The following step is the formation of formaldehyde as an intermediate, as demonstrated in the same reference by the time evolution of the products. The fate of formaldehyde may be a further reduction step to methanol or a back oxidation to formate. It was already reported that the back oxidation of HCHO to HCOOH is favored ad basic pH,^29^ while the reduction of HCHO to methanol is easier at lower pH.^30^ Furthermore, the abundance of OH^–^ under basic conditions favors the formation of oxidril radicals through the use of photogenerated holes, which may improve the oxidation of formaldehyde to formate. In addition, the liquid products formed can act themselves as hole scavengers with production of CO_2_ and H_2_ when the sulfite is fully consumed, as previously described [see refs (16 and 27)]. However, the hydrogen detected may also be the result of direct water splitting promoted by titania.^31^ However, this latter route proved ineffective without the initial activation of the reaction through the use of the sulfite and the accumulation of organic products in the liquid phase.

The best value of pH may be chosen looking at the value of the products formed and the energy stored into these compounds. Indeed, when working at pH 14, the energy content of the product, based on the heating value (HV), is greatly improved with respect to pH 7, since the formic acid has a lower HV than methanol, but this is counterbalanced by far by the increased productivity (Table 3). These results corresponded to a 0.012 and 0.022% efficiency if calculated with respect to lamp consumption for pH 7 and pH 14, respectively. Considering, instead, the measured irradiance, the efficiency increased 1 order of magnitude (0.22 or 0.40% at pH 7 and pH 14). It is clear that these results make sense only when free solar energy may be exploitable.

**Table 3:** Energy Stored into Products in the Case of Photoreaction Performed at Different pH (7 and 14)

	H_2_	CH_3_OH	HCOOH	total
LHV^a^ (kJ/mol)	241.7	639.0	209.8	–
HHV^b^ (kJ/mol)	285.9	738.2	254.6	–
energy stored (kJ/h) kg_cat_)				
pH 7 from LHV	22.2	40.3	2.1	64.6
pH 14 from LHV	11.4	0	99.4	**110.1**
pH 7 from HHV	26.3	46.5	2.5	75.3
pH 14 from HHV	13.4	0	120.7	**134.1**

a LHV = lower heating value.^32^

b HHV = higher heating value.^32^

The economic value of the products will be analyzed deeply in section 3.2.4.

#### 3.2.2 Effect of Pressure

**Figure 6:**
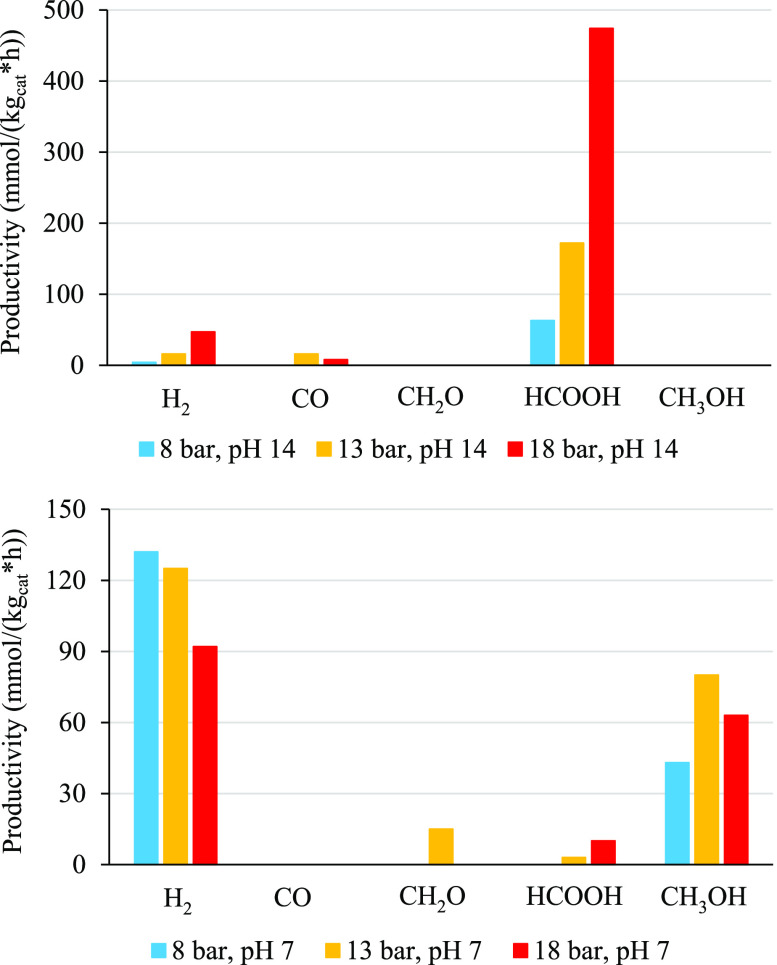
Effect of pressure on products distribution and productivity, using Au 0.2 wt %/P25 at 8, 13, and 18 bar at both neutral and basic pH. 0.6 g/L of catalyst and 0.85 g/L of HS.

Reviews on the effect of operating parameters on this reaction have highlighted the beneficial effect of temperature increase up to 80 °C and pressure higher than ambient, although very limited reports refer to significantly higher pressure.^33,34^ The data reported in Figure 6 were recorded at three different pressures (i.e., 8, 13, and 18 bar; notice that, due to sampling mode, testing should be done with a pressurized reactor, *P* > 4 bar), with a catalyst concentration of 0.6 g/L and 0.85 g/L of HS. We found that the pressure of CO_2_ mainly impacts the overall productivity when working at basic pH (14). This result is not surprising as the CO_2_ solubility increases along with pressure, leading to a higher concentration of reactant in water, to a more favorable adsorption equilibrium and, therefore, to a higher availability of the reactant over the catalyst surface.^15^ The calculated molar fraction of CO_2_ dissolved in water at 18 bar, 80 °C is ca. 0.3, ca. 0.21 at 13 bar, and ca. 0.11 at 8 bar. These values should be compared with ca. 0.03 under ambient conditions.

Negligible amounts of formaldehyde and methanol were detected at pH 14. By contrast, at neutral pH, we observed that the productivity overall decreased by ca. 1 order of magnitude, with respect to basic pH, with predominant productivity of methanol (vide supra). Despite the higher heating value of the latter, with respect to formic acid, the overall more limited productivity led to a lower stored energy than at pH 14.

Higher pressure has a negative effect on the productivity of products in the gas phase. Indeed, H_2_ productivity decreased by ca. one-third when increasing the pressure from 8 to 18 bar. In contrast, as reported above, we observed a higher reduction to methanol, whose productivity was maximum at intermediate pressure.

In all cases, the increase of pressure significantly improved the productivity of formate and, to a lower extent, of H_2_ through consecutive photoreforming at pH 14. The competitive pathway to methanol and formaldehyde at pH 7 jeopardizes the product distribution. At low pressure, formic acid is completely converted to H_2_ and methanol, whereas higher overall productivity and some formic acid and formaldehyde are accumulated at higher pressure. The maximum in methanol productivity at intermediate pressure has been already reported previously, e.g., in refs (33 and 35), and attributed to C–C coupling reactions at higher pressure.

Overall, operating at higher pressure allows one to increase the energy storage efficiency under basic pH conditions. Indeed, calculating the % of energy stored in the products, with respect to the average irradiance, one may calculate, at pH 14, a decreasing efficiency from 0.4 to 0.16 and 0.05% passing from 18 bar to 8 bar. At pH 7, instead, the operation at higher pressure is advantageous, but the more-complex product distribution jeopardizes the picture. The energy storage efficiency, with respect to available irradiance, was 0.24% at 18 bar, increasing at 0.3% at intermediate pressure and decreasing again at 8 bar (0.16%). The key in this case is the significant production of two high-energy products as H_2_ and CH_3_OH.

#### 3.2.3 Effect of Cocatalysts

Cocatalysts, whose nominal composition and metallic surface state was confirmed by XPS, can exploit different actions, depending on their nature, the method of deposition, the precursor used, etc. An effective investigation on how some of these features may affect the performance for another photosynthetic process has been recently proposed^36^ and highlights the complexity of the subject. Here, the objective is to find a material that can ensure a reasonable activity, rather than performing a detailed investigation on the effect of the cocatalysts proposed. Therefore, a phenomenological, rather than mechanistic description is proposed in the following.

Our research group found in a previous work that titania with 0.2–0.5 wt % of Au was effective for the photoreduction of CO_2_. Although this compound was prepared via deposition precipitation (DP), which ensures a good control of the size of the nanoparticles, this technique, at the moment, is not suitable for producing more than few grams of catalyst. Therefore, we decided to prepare our catalysts via wet impregnation (WI), which is a rougher but more scalable method. Furthermore, we compared different metals as cocatalysts and, to do this, it was preferable to set the atomic ratio between the moles of metal and TiO_2_ instead of the weight percentage. The latter indeed is a better quantifier to assess the cost of the material, but different metals should be compared as for their promoting effect based on the molar ratio, with respect to the semiconductor. All the cocatalysts were confirmed with a surface composition similar to the nominal one; the metallic surface state was confirmed by XPS and uniform dispersion, irrespective of the deposition and reduction methods.

**Figure 7:**
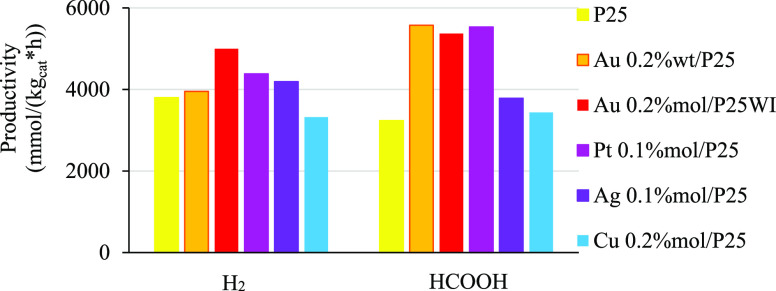
Effect of catalyst preparation and composition on products distribution and productivity at 8 bar, pH 14, 0.031 g/L of catalyst, and 1.67 g/L of HS.

0.2 wt % Au/P25 is equal to a 0.08 mol % loading; thus, as a first attempt, it was tried to improve the performance by increasing the metal loading to 0.2 mol %. According to the graph of Figure 7, this led to a 20% increase of the hydrogen productivity (5 vs 4 mol/kg_cat_ h) whereas the formate production was not affected.

In order to use a metal that was less expensive, we switched to copper, which belongs to the same group as gold but is much less expensive, keeping the same molar loading. Unfortunately, the 0.2 mol % Cu/P25 showed performance similar to the unpromoted P25, if not worse when looking at the hydrogen productivity (−13%). Other noble metals were then employed such as silver and platinum. Regarding the former, an improvement of both H_2_ and formate productivity was observed, with respect to P25; however, the performance was still below our benchmark 0.2 wt % Au/P25 (−16% H_2_ and −32% HCOOH). Lastly, the performance of platinum was quite comparable with the benchmark, despite a slightly lower hydrogen evolution.

The improvement given by gold can be ascribed to the strong electric fields created by the surface plasmonic resonance. This excites electron–hole pairs locally in titania and produces an extra number of photocatalytic reactions, which occurs at a rate several orders of magnitude higher than normal.^37^ This effect is usually visible as absorption feature in the visible range (400–600 nm for Au and Ag nanoparticles), not strictly related to our experimental conditions, but strongly depends on the size and shape of the metal particles and on the dielectric constant of the medium at the interface. For instance, a blue shift is reported, decreasing the gold particle size and with a solvent.^38^ In addition, metal deposition reduces the electron–holes recombination thanks to formation of a Schottky barrier improving the productivity. Similar considerations can be extended to platinum and silver, even if the latter has a smaller promoting effect. On the other hand, copper did not seem to add advantages, if compared to bare P25, although the Cu-promoted sample was characterized by a narrower band gap (Table 2), which may be an important parameter to promote the reaction through sunlight in perspective.

A systematic investigation on the effect of metal cocatalysts for TiO_2_ has been performed for a very similar application, i.e., photocatalytic water splitting.^36^ The effect of various metals on structural, textural and optical properties has been considered. The main observed effect was the variation of the band gap, since the other properties were not significantly or systematically changed by the addition of the cocatalyst. However, the decrease of band gap observed was not in line with the scale of activity. By considering all the parameters, the authors observed that the activity trend fits correctly the work function of the metal, namely Pt (5.93 eV), Pd (5.60 eV), Cu (5.10 eV), Ru (4.71 eV), and Ag (4.26 eV). The H_2_ increased with increasing metal work function due to slower rate of charge recombination. Pt was a better cocatalysts for TiO_2_ due to bigger work function and upward-bent band, that let the photogenerated electrons remain trapped in metal sites. Ag and Cu were less due to downward-bent band gaps that allow the electrons to move back to TiO_2_ (faster charge recombination). Similar interpretation can be applied here.

As a last point, metal reduction was achieved in different ways, by chemical reduction when loading the metals by deposition precipitation or by reduction in H_2_ when using impregnation. Different reduction temperatures (based on TPR analysis) have been used that may affect the properties of titania. However, the characterization did not show important structural modifications of titania. What is most important is the partial reduction of the titania, which may occur under hydrogen atmosphere and be catalyzed by the metal itself. The different reduction method is due to the need of high dispersion at higher loading, unachievable by impregnation but only through sol colloidal deposition.

**Figure 8:**
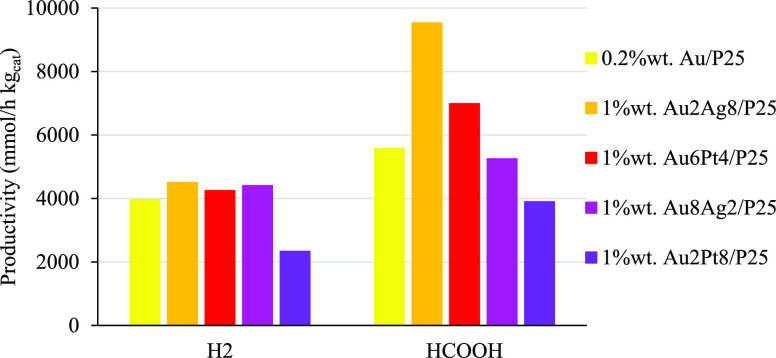
Effect of bimetallic catalyst on products distribution and productivity at 8 bar, pH 14, 0.031 g/L of catalyst, and 1.67 g/L of HS.

The next step was to combine the effect of different metals over the same catalyst. To do this, we selected the dp as the preparation method to guarantee intimate contact between the metals and high dispersion, adding to Au (0.2 wt %) a second metal, Pt or Ag (0.8 wt %). The results are reported in Figure 8. Looking at the performance of monometallic materials, we expected to achieve better productivity using Au+Pt or possibly by further increasing the Au loading (0.8 wt %). In contrast, it was found that a relatively low loading of Au was enough to promote the reaction, without significant auxilium from the increase of Au loading or the addition of Pt. On the contrary, a synergistic effect between Au and Ag was achieved. Indeed, the best results were obtained with Au_2_Ag_8_ 1 wt %, since the formate production was doubled with respect to the benchmark, while the similar catalyst loaded with Pt instead of Ag showed a 30% reduction of the performance. All in all, while the H_2_ productivity remained constant, the effect of the second cocatalyst varied remarkably the formate productivity. If the addition of a small amount of Ag (0.2%wt) did not overperform the benchmark activity, at the expenses of a considerable increase of the more expensive Au metal, the addition of Pt slightly enhanced the formate production rate.

In every case, a full conversion of the inorganic hole scavenger was achieved. Thus, HS concentration was increased in order to boost the conversion of CO_2_ to formate. The test was performed with the most active photocatalyst, which is the 1% Au_2_Ag_8_/P25, and by employing a higher amount of hole scavenger (i.e., 6 g/L). The results in terms of hydrogen production were encouraging, since a productivity of 20 000 mmol/h kg_cat_ was achieved while that of HCOOH reached 26 000 mmol/h kg_cat_; however, we observed, once more, the full consumption of HS during the standard 24 h test, even by increasing the HS concentration.

The correlation between products distribution, reaction time, and the conversion of the HS has been already discussed in a previous work using bare TiO_2_ P25:^21^ until the complete conversion of the sulfite (depending on its concentration and reaction time), only liquid-phase products have been obtained. Furthermore, a consecutive reaction pathway has been observed (depending on pH and visible only under neutral conditions) for the progressive conversion of formic acid to formaldehyde and methanol.

To further deepen this point, we have reduced the testing time with the most interesting 1% Au_2_Ag_8_/P25 catalyst. At pH 14 and a pressure of 18 bar, only HCOOH was obtained (9220 mmol/kg_cat_ h after 3 h of irradiation and 18 247 mmol/kg_cat_ h after 6 h), leading to 15.9% conversion of the HS after 3 h and 46.5% after 6 h, strongly overperforming all the previously reported results. The consumption of all the HS leads to the progressive conversion of the formed organic molecules, acting themselves as hole scavengers when the sulfite is no longer present.

#### 3.2.4 Economic Considerations

Since our goal is to design a technology that is effective and sustainable, it is necessary to determine the catalyst with the best performance/price ratio, as well the optimal conditions that lead to the most valuable chemicals. Regarding the latter, it was found that it is a great undertaking to increase the HCOOH productivity by working at pH 14, since its selling value is ∼0.5 €/kg for 85 wt % in water^32^ (27 €/kmol) vs 0.3 €/kg for methanol^33^ (>95 wt %, ca. 10 €/kmol) and 0.7 €/kg (0.7 €/kmol) for hydrogen. Indeed, the productivity of HCOOH at high pH is between 2-fold to 8-fold greater than that of methanol under neutral conditions, thus leading to a higher potential profit.

The value of the photocatalyst is important as well. Tables 4 and 5 summarize the raw costs that may be hypothesized for each catalyst, based on its constituents price. This item is considered significant and variable, depending on the formulation, the selected metal, and its loading. The investment for the photoreactor is not computed at the moment, but it is considered equal independently of the catalyst; therefore, for comparative purposes, it is neglected. The relative revenues from selling formic acid and hydrogen, detected as only products under basic pH, were computed. No additional separation is foreseen at the moment besides the recovery of the gas and the liquid products, but again, the technology will be similar for all the different catalyst formulations compared here. Similarly, the utilities and installation costs for compression will increase with increasing the operating pressure, except if using a CO_2_ storage facility where the reactant is already compressed. Because of all these uncertainties, this aspect is not currently computed, leaving its estimation to the definition of a more precise case history.

**Table 4:** Costs of Catalysts Constituents^a^

	cost €/kg
compound	
P25	230 €/kg
Cu	6.20 €/kg
Ag	560 €/kg
Au	48 870 €/kg
Pt	29 770 €/kg
energy (including service costs)	0.10 €/kWh
hydrogen	1.41 €/kmol
formate	27.16 €/kmol

a Prices of metals obtained from official quotations of London Metal Exchange (updated Dec. 24, 2019). Cost of energy determined by Servizio Elettrico Nazionale S.p.A.^39^.

**Table 5:** Gross Profit Per Each Kilogram of Catalyst Employed for One Year of Production (24/7) in the Hypothesis of UV Light Irradiation

catalyst	catalyst raw cost (€/kg)	formate productivity (kmol/(yr kg_cat_))	hydrogen productivity (kmol/(yr kg_cat_))	total selling value (€/yr)
Au 0.2 wt %/P25	327.74	48.93	34.67	1377.81
Cu 0.2 mol %/P25	230	30.16	29.14	860.23
Au 0.2 mol %/P25	**474.35**	47.08	**43.85**	1340.56
Ag 0.1 mol %/P25	230.77	33.14	36.74	951.93
Pt 0.1% mol/P25	303.83	48.49	38.40	1382.04
Au2Ag8 1 wt %/P25	332.22	**83.56**	39.46	**2325.18**

At first, it is clearly visible that the most inexpensive functionalized catalyst is the one loaded with copper, but unfortunately it is even the less active. All the catalysts added with gold increased the productivity significantly, with respect to P25; however, the costs of the materials is almost doubled if compared with bare P25, notwithstanding the very small metal loading, and reduces the profitability of this technology. Silver and platinum seem to be a good compromise if looking only at the monometallic deposited catalyst. Anyway, the results of the calculations are that Au_2_Ag_8_ is the compound with the best performance/price ratio. The positive difference between revenues and costs, even though this very rough preliminary assessment, opens the way to a more-detailed design and optimization of the process.

Comparison of the economics with rival technologies is hard, both for the current state of development of photoreduction and for the very different scale envisaged. An estimation of the plant costs for the electroreduction of CO_2_ (as bicarbonate or gas) to CO or formate with a scale of 100 t/day of product has been recently reported.^36^ An investment of 6–14 million dollars is indicated for the construction of the electrolyzer and payback time estimated between 1 and 3 years. The main cost is, of course, that of electricity, with strong sensitivity on this item. The alternative of power-to-gas valorization of CO_2_ to produce synthetic methane has been also estimated. Currently, the cost of the electrolyzer and of power are the limiting factors, that make the cost of methane (8–43 $/kWh) not yet competitive with that of natural gas.^36^

## 4 Conclusions

The photoreduction of CO_2_ has been studied operating under different conditions and investigating the effects of catalyst formulation. A comparison between different techniques for loading the cocatalyst (wet impregnation, deposition–precipitation) was done as well.

pH was found to play a major role in the product distribution. On one hand, neutral pH seems to favor the production of hydrogen and methanol, while basic pH enhances the conversion and the productivity of formic acid, besides hydrogen. The latter is due to the full consumption of the sulfite used as a hole scavenger. Indeed, tests with limited HS conversion overperformed the results at each pH tested, preventing the consumption of the organics formed in liquid phase and leading to negligible gas-phase products.

Moreover, the productivity increases along with pressure, especially at basic pH, because of the increase of reactants concentration in the liquid phase.

Among the monometallic promoted catalysts, the ones loaded with Au and Pt gave larger improvement, in terms of productivity, with respect to bare P25, because of the efficient electron drainage, which prevents the electron–hole recombination. In contrast, Ag and Cu deposition produced smaller benefits even if Cu may be interesting for photoreduction under solar light as its band gap is only 2.71 eV and its cost is negligible in catalyst formulation.

Bimetallic catalysts led to very promising results, in particular Au_2_Ag_8_ 1 wt %/P25, and may help with reducing the costs of catalyst combining valuable metals with less-expensive ones through a considerable improvement of productivity.
